# Sad Dad: An Exploration of Postnatal Depression in Fathers

**DOI:** 10.1192/pb.bp.114.049577

**Published:** 2016-06

**Authors:** Floriana Coccia

This book sets out to make readers aware of the evidence and opinions regarding postnatal depression in fathers. The first chapter gathers together the published evidence available. This is followed by several chapters of interviews with experts in the field, predominantly psychotherapists. There are chapters on the role of fathers, single-sex parents and some personal experiences of men who have had depression related to the birth of their children.

There is not very much evidence yet available and very few of the studies look at postnatal depression in isolation. Many of them include depressive episodes that occur in men while their partners are pregnant and some studies also include anxiety symptoms. Apart from research arising from the Avon Longitudinal Study of Parents and Children, there is little information given on the size of the studies, making it difficult to assess the validity without looking for the individual paper. One of the interesting references in this section was on the anxiety experienced by men related to childbirth and how this can have an impact on their postnatal well-being.

The subsequent chapters are the views of individual psychotherapists who see men with depression. Here there is an attempt to describe how men present differently in depressive episodes than do women and that avoidance of the home by working long hours, alcohol use and risky behaviours are more common. There is reference to ‘several’ or ‘many’ patients that the psychotherapists have seen on which they base their views and I question whether this is then generalisable in clinical practice.

The final chapters are more a narrative by the author looking into other elements related to fathers and the role of men in parenting. There is some thought given to how men adapt to childbirth and how they are sometimes excluded in the process that is largely focused on mother and infant. I found that this was somehow belittling to men, suggesting that they were unable to manage the demands put on them and making them seem somehow fragile.

It is difficult to suggest who the book is aimed at. I think that healthcare professionals would find the heavy slant towards opinion unhelpful. Fathers may find some of the psychodynamic language a bit hard to relate to. Olivia Spencer has made a start at getting people to think a little bit more about including fathers in childbirth and the surrounding months, but I remain unconvinced that she has made a case for postnatal depression in fathers.

